# Machine Learning for the Detection and Segmentation of Benign Tumors of the Central Nervous System: A Systematic Review

**DOI:** 10.3390/cancers14112676

**Published:** 2022-05-27

**Authors:** Paul Windisch, Carole Koechli, Susanne Rogers, Christina Schröder, Robert Förster, Daniel R. Zwahlen, Stephan Bodis

**Affiliations:** 1 Department of Radiation Oncology, Kantonsspital Winterthur, 8400 Winterthur, Switzerland; carole.koechli@uzh.ch (C.K.); christina.schroeder@ksw.ch (C.S.); robert.foerster@ksw.ch (R.F.); daniel.zwahlen@ksw.ch (D.R.Z.); 2 Department of Radiation Oncology, Kantonsspital Aarau, 5001 Aarau, Switzerland; susanne.rogers@ksa.ch (S.R.); stephan.bodis@ksa.ch (S.B.)

**Keywords:** machine learning, deep learning, benign brain tumor, vestibular schwannoma, meningioma, pituitary adenoma

## Abstract

**Simple Summary:**

Machine learning in radiology of the central nervous system has seen many interesting publications in the past few years. Since the focus has largely been on malignant tumors such as brain metastases and high-grade gliomas, we conducted a systematic review on benign tumors to summarize what has been published and where there might be gaps in the research. We found several studies that report good results, but the descriptions of methodologies could be improved to enable better comparisons and assessment of biases.

**Abstract:**

**Objectives**: To summarize the available literature on using machine learning (ML) for the detection and segmentation of benign tumors of the central nervous system (CNS) and to assess the adherence of published ML/diagnostic accuracy studies to best practice. **Methods**: The MEDLINE database was searched for the use of ML in patients with any benign tumor of the CNS, and the records were screened according to PRISMA guidelines. **Results**: Eleven retrospective studies focusing on meningioma (n = 4), vestibular schwannoma (n = 4), pituitary adenoma (n = 2) and spinal schwannoma (n = 1) were included. The majority of studies attempted segmentation. Links to repositories containing code were provided in two manuscripts, and no manuscripts shared imaging data. Only one study used an external test set, which raises the question as to whether some of the good performances that have been reported were caused by overfitting and may not generalize to data from other institutions. **Conclusions**: Using ML for detecting and segmenting benign brain tumors is still in its infancy. Stronger adherence to ML best practices could facilitate easier comparisons between studies and contribute to the development of models that are more likely to one day be used in clinical practice.

## 1. Introduction

Whilst an increase in computational power and the development of more user-friendly software libraries have accelerated the adoption of machine learning (ML) techniques in both neuro-radiology and neuro-oncology, much of the research that is being published focuses on malignant tumor entities, such as high-grade gliomas or brain metastases [1].

A possible explanation for this phenomenon lies in the availability of data that are required to train, validate and test ML models. While almost any hospital will have a sufficient number of cases for epidemiologically significant entities such as brain metastases, this is not the case for central nervous system (CNS) tumors with lower incidences, such as many benign brain tumors [2].

In addition, most publicly available imaging datasets are comprised of malignant entities. The popular Brain Tumor Segmentation (BraTS) Challenge dataset consists exclusively of gliomas, which also applies to most of brain datasets that are available as part of The Cancer Imaging Archive (TCIA) [3,4].

Despite these obstacles, there have been publications investigating the use of ML for benign CNS tumors [5,6]. This review will therefore summarize the research that has been conducted on this topic in systematic fashion and assess the quality of the studies that have used ML for tumor detection and segmentation, as has been done previously for malignant CNS tumors [7,8]. The goal is to create a point of reference that other researchers can use to identify gaps in the research that are worthy of further investigation and to identify possible shared issues regarding methodologies or their descriptions so that they can be addressed by future publications.

While there are numerous potential benefits to having machine learning techniques taking over parts of the radiology workflow or serving as automated second opinions, this requires common reporting standards to identify approaches that are worthy of being pursued further with the goal of possibly translating them into routine clinical practice someday.

## 2. Methods

### 2.1. Literature Search

The review was conducted according to the Preferred Reporting Items for Systematic Reviews and Meta-Analyses (PRISMA) guidelines [9]. Studies published in English after 2000 that used any kind of machine learning technique for the detection or segmentation of benign tumors of the CNS were included. Studies using semi-automatic segmentation requiring manual user input prior to creating the segmentation were not included. Since studies using segmentation only as a means to predict clinical or pathologic features frequently provide little detail on the segmentation methodology, so these studies were not included as well. No limits regarding size of the patient collective or length of follow-up were applied.

The Medical Literature Analysis and Retrieval System Online (MEDLINE) database was searched on 14 June 2021 via the PubMed interface. The query was designed to include all studies that contained one or more words from two groups, one group comprised of words that indicate the usage of an ML technique (automated, computer aided, computer-aided, CAD, radiomic, texture analysis, deep learning, machine learning, neural network, artificial intelligence) and the other group comprised of words associated with benign brain tumors (meningioma, meningiomas, schwannoma, schwannomas, craniopharyngioma, craniopharyngiomas, ganglioglioma, gangliogliomas, glomus, pineocytoma, pineocytomas, pilocytic, pituitary, benign brain tumor, benign brain tumors).

The complete search query that was used was therefore:

“((automated[title]) OR (computer aided[title]) OR (computer-aided[title]) OR (CAD[title]) OR (radiomic[title]) OR (radiomics[title]) OR (texture analysis[title]) OR (texture analyses[title]) OR (textural analysis[title]) OR (textural analyses[title]) OR (deep learning[title]) OR (machine learning[title]) OR (ML[title]) OR (neural network[title]) OR (NN[title]) OR (artificial intelligence[title]) OR (AI[title])) AND ((meningioma[title]) OR (meningiomas[title]) OR (schwannoma[title]) OR (schwannomas[title]) OR (craniopharyngioma[title]) OR (craniopharyngiomas[title]) OR (ganglioglioma[title]) OR (gangliogliomas[title]) OR (glomus[title]) OR (glomera[title]) OR (pineocytoma[title]) OR (pineocytomas[title]) OR (pilocytic[title]) OR (pituitary[title]) OR (benign brain tumor[title]) OR (benign brain tumors[title]) OR (benign brain tumour[title]) OR (benign brain tumours[title])) AND (“2000/01/01”[Date-Create]: “2021/06/14”[Date-Create])”

The review had neither been registered nor had a protocol published beforehand.

After exclusion of duplicates, the titles and abstracts were screened, and only relevant publications proceeded to full-text screening. The decision as to whether a study met the inclusion criteria of the review was performed by two authors (P.W. and C.K.) without the use of automated tools. A third author (C.S.) acted as a referee in case of a potential disagreement between the two authors responsible for screening. All articles that did not focus on the use of ML for detection or segmentation in patients with benign brain tumors were excluded. 

### 2.2. Data Extraction

Two authors (P.W. and C.S.) independently extracted data and discussed any discrepancies. Data were extracted with regard to:

1. Study parameters: authors, title, year, design, number of patients in training/test set, ground truth, inter-/intrarater variability, task, conflict of interest, sources of funding.
2. Clinical parameters: tumor entity, tumor volume, treatment of tumors prior to imaging.
3. Imaging parameters: MRI machine, field strength, slice thickness, sequences.
4. ML parameters: algorithm, dimensionality, training duration and hardware, libraries/frameworks/packages, data augmentation, performance measures, explainability/interpretability features, code/data availability.

## 3. Results

**Figure 1:**
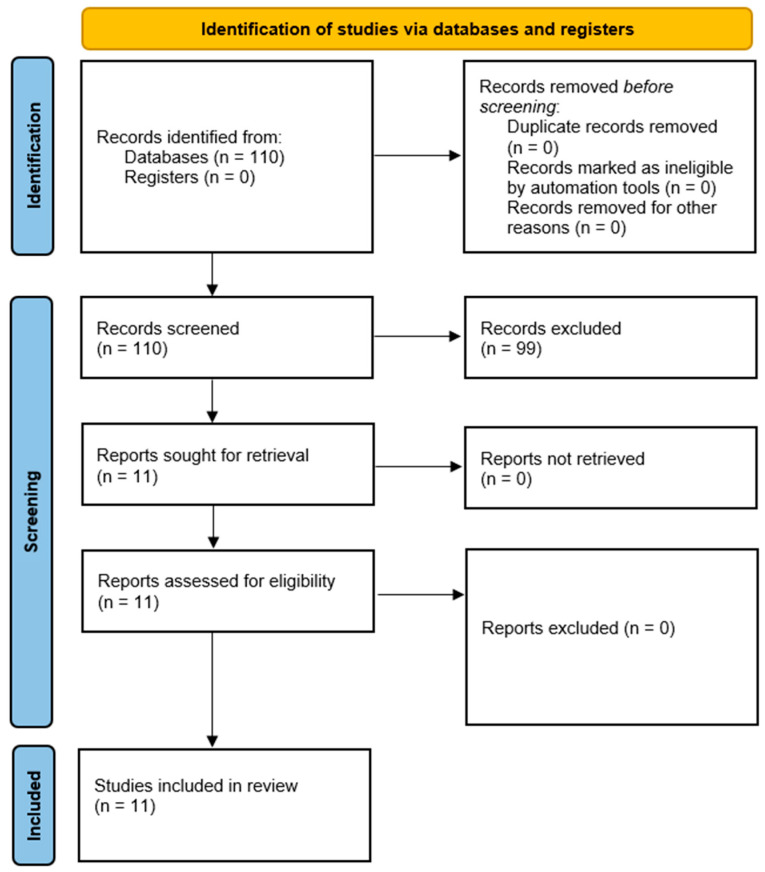
Inclusion workflow diagram according to PRISMA 2020. From: Page MJ, McKenzie JE, Bossuyt PM, Boutron I, Hoffmann TC, Mulrow CD, et al. The PRISMA 2020 statement: an updated guideline for reporting systematic reviews. BMJ 2021;372:n71. Doi: 10.1136/bmj.n71. For more information, visit: http://www.prisma-statement.org/ (accessed on 25 April 2022).

The inclusion workflow is depicted in Figure 1. The query returned 110 publications and no duplicates. When screening the records, 99 articles were excluded. A complete list of the excluded articles and the respective reasons for exclusion is provided in Supplementary Table S1. Thirty three articles were excluded due to predicting only pathological features, e.g., grade (n = 16), or differentiating between tumor entities (n = 8) [10,11,12,13,14,15,16,17,18,19,20,21,22,23,24,25,26,27,28,29,30,31,32,33,34,35,36,37,38,39,40,41,42]. Thirty four articles were excluded due to predicting only clinical parameters, e.g., tumor consistency (n = 7), response/treatment outcome (n = 12) or brain/bone invasion (n = 4) [43,44,45,46,47,48,49,50,51,52,53,54,55,56,57,58,59,60,61,62,63,64,65,66,67,68,69,70,71,72,73,74,75]. Twelve articles did not focus on ML techniques [76,77,78,79,80,81,82,83,84,85,86,87]. Eight articles were not original reports but reviews or editorials [88,89,90,91,92,93,94,95]. Three articles used semi-automatic segmentation techniques [96,97,98]. Three articles dealt with the application of ML techniques to brain tumors in dogs [99,100,101]. Six articles were excluded for other reasons, such as using ML for image reconstruction (n = 1), analyzing tissue (n = 3) or non CNS-tumor entities (n = 1) and predicting drivers of costs (n = 1) [102,103,104,105,106]. 

All eleven articles that underwent full text screening were included, and the extracted characteristics from all articles are provided in Supplementary Table S2. All studies were conducted retrospectively and published between 2018 and 2021. The tumor entities that were investigated were meningioma (n = 4), vestibular schwannoma (n = 4), pituitary adenoma (n = 2) and spinal schwannoma (n = 1). Between 50 and 1876 patients were used for developing or testing the models in the respective studies. Notably, only the study by Qian et al. studied tumor detection using healthy controls or controls with other cerebral neoplasms [107]. Other studies claiming to do tumor detection did so by doing tumor segmentation, but these models never had to consider the possibility that a tumor was not present. 

### 3.1. Disclosures and Declarations

The authors of two publications uploaded code to a public repository that was referenced in the manuscript [108,109]. The remaining publications did not mention code availability. No data were shared, but two articles mentioned the option to obtain data from the corresponding author upon request [109,110]. Employment by Philips was the most frequent conflict of interest at study level and declared in two publications [111,112]. A patent application related to the published work was present in one publication. Six publications explicitly stated that the authors had no conflict of interest. The Ministry of Science and Technology of Taiwan was the most frequent source of funding (n = 2), and three publications stated no additional funding [108,113]. 

### 3.2. Imaging

All studies used magnetic resonance imaging (MRIs) with a field strength of between one and three Tesla for imaging. Whilst six studies used homogenous datasets from a single device, the remaining studies used datasets consisting of images from multiple devices. Where reported, slice thickness was between one and six millimeters. All studies used a T1-weighted sequence, presumably with contrast enhancement, though this was not explicitly stated in all publications. T2-weighted sequences were used in seven studies, and two specified the use of a T2 FLAIR sequence.

### 3.3. Ground Truth

All manuscripts claimed that at least two people worked on tumor segmentation. Additional information was frequently lacking—for example, whether every image was independently annotated by two people and whether the annotators had access to clinical information. Similarly, only four manuscripts reported interrater variability. The most frequently used metric for interrater variability, the dice coefficient, was between 0.89 and 0.94 [5,112,114,115].

### 3.4. Modeling

All publications used convolutional neural networks for modeling. Eight publications used a designated test set, unseen by the model during training, and one of which can be considered an external test set, as the images were provided by an institution other than the one that supplied the training set. The publications that did not use a separate test set used cross-validation or the contours of one annotator for training and those of the other for testing.

Regarding libraries, five publications mentioned the use of tensorflow and one the use of PyTorch. The remaining publications did not reference libraries in the manuscript. The use of data augmentation was mentioned in three publications. The implementation of explainability features was not discussed, but one publication analyzed the performance of the classifier depending on tumor volume [5].

### 3.5. Meningioma

All publications on meningiomas (n = 4) used meningiomas from different intracranial locations. 

Laukamp et al. published two articles on meningioma segmentation [111,112]. For the first publication, they trained a network based on the DeepMedic architecture with contrast-enhanced T1 (T1c) and T2FLAIR images from glioblastoma cases and used those to segment meningiomas, which resulted in a Dice coefficient of 0.78. In their second publication, they used grade I/II meningiomas for training as well and tested on the same cohort, this time achieving a dice coefficient of 0.91 for the contrast-enhancing tumor. 

Zhang et al. used T1c slices from 1876 patients to train a model to segment meningiomas and predict the tumor grade by using the segmentation [109]. To describe the performance of their segmentation, they used a less established concept called “tumor accuracy” defined as the percentage of correctly predicted pixels in the tumor, which was 0.814.

Bouget et al. used T1c MRIs of 698 patients with various architectures, the best of which achieved a dice coefficient of 0.73 for meningioma segmentation. Notably, the authors used one fold of the cross-validation for testing, rather than a separate set with previously unseen data [5].

### 3.6. Schwannoma

Shapey, Wang et al. used T1c and T2 images from 243 patients to train a model to segment vestibular schwannomas. Median tumor size in the test set was 1.89 mL and the best dice coefficient was 0.937 [115].

George-Jones et al. analyzed a cohort of 65 patients with a median tumor volume of only 0.28 mL [114]. Unlike other publications, the authors did not report dice coefficients, but instead tried to analyze how well the model was able to detect growth compared to the manual segmentations which were used as the ground truth. The model achieved an area under the receiver operating characteristic curve (ROC-AUC) of 0.822.

Lee et al. published two manuscripts on vestibular schwannoma segmentation and used the segmentations to analyze changes in volume [108,113]. The authors achieved a dice coefficient of 0.90 when taking advantage of both T1c- and T2-weighted imaging data.

Ito et al. used a dataset of 50 patients for bounding-box segmentations of spinal schwannomas, and used one cross-validation fold for testing instead of a fully independent test set [6]. The authors reported an accuracy of 0.935, though the actual ground-truth was not explicitly stated.

### 3.7. Pituitary Adenoma

Qian et al. published a study on pituitary adenoma detection, and it is the only study included in this manuscript that used a control group of patients without tumors [107]. The reported overall accuracy was 0.91. 

Wang, Zhang et al. used a collective of 163 patients to train and test automated segmentation for pituitary adenomas [110]. The dice coefficient for all slices of the tumors was 0.898. 

Highlighted study, imaging and machine learning parameters can be found in Table 1, Table 2 and Table 3 respectively.

## 4. Discussion

The results of our review indicate that machine learning for the segmentation, and even more so for the detection, of benign brain tumors is still in its infancy but is gaining traction. 

All included studies were published after 2018 and used deep learning, which is in line with the finding by Cho and colleagues, who found a shift from classical ML to deep learning for brain metastasis detection after 2018 [7].

Guidelines and checklists for diagnostic accuracy, and artificial intelligence studies, have been available for some time [8,116]. The fact that all studies mentioned two physicians being involved with creating the ground truth can be considered as evidence that the authors were aware of at least some of their requirements and best practices. However, many studies were vague about other items of these guidelines, or did not mention them at all, even though they would apply, which indicates that the guidelines are only enforced to a limited degree when a manuscript is reviewed prior to publication.

Many questions regarding the exact methodology could be answered by providing the code that was used for the project, but a public repository was only referenced in two of the included studies. Data sharing is even rarer, though this is somewhat understandable given the sensitive nature of complete cranial MRI datasets that could be used for face recognition if the resolution is sufficiently high [117].

As an example, the study by Qian et al. mentions that the data were augmented and then divided “into training or testing set in a ratio of 8:2 for further analysis”. This makes it seem like slices from the same patient could have been present in the training and test sets, and maybe even different augmentations based on the exact same slice could have been present in the training and test sets. If this was the case, it is likely that the performance of the model would be attributable to overfitting and unlikely to be sustained on previously unseen data [107]. If code had been provided, this could have been easily clarified by any technical reader or reviewer.

In general, the presence of overfitting cannot really be assessed for the majority of publications, as external test sets were almost never used. The logistics involved with obtaining data for fairly rare tumor entities from other institutions is challenging, but strategies to mitigate this could be employed. If a hospital has more than one MRI machine, one might, for example, use data from one machine for testing and data from the other machines for training. 

Considering the progress in services that allow researchers to deploy their models, we might get to a point where researchers host models so that reviewers/readers can test them with their own data in the future to make additional conclusions regarding generalizability. 

The fact that only one publication tried to train a network for detection is surprising, as overlooking a small, benign brain tumor on an MRI is a real-world problem that could be addressed by having an artificial intelligence function as a safety net. This would, however, require a low false positive rate of the AI to not increase the radiology workload, and the logistics of creating and processing datasets with different tumors, including healthy controls, remain a hurdle.

Creating a dataset for segmentation is easier, as it only requires images of patients with the tumor one is trying to segment. Automatic segmentation has a clear application, as fractionated and stereotactic radiotherapy are treatment options for many benign tumors of the CNS and require segmentation of the tumor prior to treatment. Furthermore, volumetric measurements, to determine if a tumor is growing, could be facilitated by automatic segmentations [118,119].

Considering that benign brain tumors are relatively rare, and that related datasets are consequently often small, it was surprising to see that only three publications reported the use of data augmentation techniques, as they are an effective way to add heterogeneity to the data [120]. One underlying reason might be that such techniques are less established for 3D convolutional neural networks (CNNs), which were used in several publications [121]. 

Lastly, the use of explainability/interpretability features could be expanded. Implementing model explainability has the potential to not only enable trust in and adoption of models by physicians, but also to support the training process by discovering pitfalls regarding data selection and overfitting [122].

As a general consideration, it will be interesting to see if an automated detection of benign brain tumors, if feasible, actually improves outcomes. As other studies on screening, even in malignant entities show that finding more tumors is not necessarily a guarantee for making patients’ lives better and longer, which should always be the ultimate goals. This question, however, can only be answered by enrolling patients in a randomized-controlled trial testing ML-augmented radiology vs. non-ML-augmented radiology once the technique has matured.

Limitations of this review include the fact that studies using segmentation only as a means for predicting clinical or pathologic features were not considered. In addition, the small sample size and heterogeneous descriptions of methodologies prevent us from drawing quantitative conclusions. Only one database was queried, but this limitation was mitigated by the fact that the majority of publications in the field of ML for radiology appear in PubMed-indexed journals.

Strengths of this review include the adherence to PRISMA guidelines and the fact that systematically searching the literature showed several ways to easily improve the quality of publications in the future—e.g., ensuring code availability. We hope that our review will also serve as a starting point for interested ML researchers to identify interesting topics in the field of benign brain tumors more efficiently by getting up to speed with the literature more quickly.

## 5. Conclusions

In conclusion, machine learning for detecting and segmenting benign tumors of the CNS is gaining traction but is still at an early stage. The possible presence of overfitting and other biases in several publications makes it difficult to assess whether the high dice coefficients that were reported would be achievable when deploying the models on data from other institutions. Enforcing guidelines at the review and publication level could enhance the quality of published studies. This is likely to happen as ML in medicine becomes more established and those involved in the publication process become increasingly aware of the possible pitfalls.

## Figures and Tables

**Table 1:** Study and clinical parameters.

Author	Year	Tumor Entity	Average Tumor Volume	No. of Patients
Wang, Zhang et al. [110]	2021	Pituitary adenoma	7.9 mL	163
Bouget et al. [5]	2021	Meningioma	29.8 mL (surgically resected); 8.47 mL (untreated)	698
Lee et al. [108]	2021	Vestibular schwannoma	2.05 mL	381
Ito et al. [6]	2020	Spinal schwannoma	Not mentioned	50
Ugga et al. [89]	2020	Meningioma	Not mentioned	1876
Lee et al. [113]	2020	Vestibular schwannoma	Not mentioned	516
George-Jones et al. [114]	2020	Vestibular schwannoma	0.28 mL	65
Qian et al. [107]	2020	Pituitary adenoma	Not mentioned	149
Laukamp et al. [112]	2020	Meningioma	∼31 mL	126
Shapey, Wang et al. [115]	2019	Vestibular schwannoma	1.89 mL (test set)	243
Laukamp et al. [111]	2018	Meningioma	30.9 mL	56 (test set)

**Table 2:** Imaging parameters.

Author	Field Strength [T]	Slice Thickness [mm]	MRI Sequence Used for Task
Wang, Zhang et al. [110]	3	3	T1c
Bouget et al. [5]	1.5/3	heterogeneous	T1c
Lee et al. [108]	1.5	3	T1c; T2
Ito et al. [6]	1.5/3	heterogeneous	T1; T2
Ugga et al. [89]	3	5	T1c
Lee et al. [113]	1.5	3	T1; T1c; T2
George-Jones et al. [114]	1.5/3	heterogeneous (median 3.3)	T1c
Qian et al. [107]	1.5	3	T1; T2
Laukamp et al. [112]	1–3	heterogeneous	T1c; T2FLAIR
Shapey, Wang et al. [115]	1.5	1.5	T1c; T2
Laukamp et al. [111]	1–3	1–6	T1c; T2FLAIR

**Table 3:** Machine learning parameters.

Author	Detection/Segmentation Algorithm	Data Augmentation	Performance Measures	Explainability/Interpretability	Code Availability	Data Availability
Wang, Zhang et al. [110]	Convolutional Neural Network (Gated-Shaped U-Net)	Not mentioned	Dice coefficient: 0.898	Not mentioned	Not mentioned	From authors upon request
Bouget et al. [5]	Convolutional Neural Network (3D U-Net, PLS-Net)	Horizontal and vertical flipping, random rotation in the range [−20°, 20°], translation up to 10% of the axis dimension, zoom between [80, 120]%, and perspective transform with a scale within [0.0, 0.1]	Best dice coefficients: 0.714 (U-Net), 0.732 (PLS-Net)	Authors analyzed the influence of tumor volume on the performance of the classifiers	Not mentioned	Not mentioned
Lee et al. [108]	Convolutional Neural Network (Dual Pathway U-Net Model)	Not mentioned	Dice coefficient: 0.9	Not mentioned	https://github.com/KenLee1996/Dual-pathway-CNN-for-VS-segmentation (accessed on 25 April 2022)	Claims that all data is in the supplement but that appears not to be the case
Ito et al. [6]	Convolutional Neural Network (YOLO v3)	Random transformations such as flipping and scaling	Accuracy: 0.935	Not mentioned	Not mentioned	Not mentioned
Ugga et al. [89]	Convolutional Neural Network (Pyramid Scene Parsing Network)	Not mentioned	Tumor accuracy: 0.814	Not mentioned	https://github.com/zhangkai62035/Meningioma_demo (accessed on 25 April 2022)	From authors upon request
Lee et al. [113]	Convolutional Neural Network (Dual Pathway U-Net Model)	Not mentioned	Dice coefficient: 0.9	Not mentioned	Not mentioned	Not mentioned
George-Jones et al. [114]	Convolutional Neural Network (U-Net)	Not mentioned	ROC-AUC: 0.822 (for agreement wether a tumor had grown between scans)	Not mentioned	Not mentioned	Not mentioned
Qian et al. [107]	Convolutional Neural Networks (one per combination of perspective/sequence)	Zooming (0–40%), rotating (−15° to +15°), and shear mapping (0–40%)	Accuracy: 0.91	Not mentioned	Not mentioned	Not mentioned
Laukamp et al. [112]	Convolutional Neural Network (DeepMedic)	Not mentioned	Dice coefficient: 0.91	Not mentioned	Not mentioned; DeepMedic is a public repository	Not mentioned
Shapey, Wang et al. [115]	Convolutional Neural Network (U-Net)	Not mentioned	Dice coefficient: 0.937	Not mentioned	Not mentioned	Not mentioned
Laukamp et al. [111]	Convolutional Neural Network (DeepMedic)	Not mentioned	Dice coefficient: 0.78	Not mentioned	Not mentioned; DeepMedic is a public repository	Not mentioned

## Supplementary Materials

The following supporting information can be downloaded at: https://www.mdpi.com/article/10.3390/cancers14112676/s1, Table S1: Excluded studies, Table S2: Study parameters.
