# Supplementary material for: Machine Learning for the Detection and Segmentation of Benign Tumors of the Central Nervous System: A Systematic Review
Source: Cancers (Basel). 2022 May 27;14(11):2676. doi: 10.3390/cancers14112676 (PMC9179850; doi:10.3390/cancers14112676)
Supplement: Supplementary file 1 [file cancers-14-02676-s001.zip › Supp_Table S1.pdf]

Authors	Title	Year	Reason for Exclusion
Zhai et al.	Preoperative Prediction of Meningioma Consistency via Machine Learning-Based Radiomics	2021	Predicting clinical parameters (consistency)
Huang et al.	Machine Learning-Based Multiparametric Magnetic Resonance Imaging Radiomic Model for Discrimination of Pathological Subtypes of Craniopharyngioma	2021	Predicting pathologic features (subtype)
Ko et al.	Pre-operative MRI Radiomics for the Prediction of Progression and Recurrence in Meningiomas	2021	Predicting clinical parameters (recurrence, progression)
Shapey et al.	Artificial Intelligence Opportunities for Vestibular Schwannoma Management Using Image Segmentation and Clinical Decision Tools	2021	No original article ('news')
Xiao et al.	Three-Dimensional Radiomics Features From Multi-Parameter MRI Combined With Clinical Characteristics Predict Postoperative Cerebral Edema Exacerbation in Patients With Meningioma	2021	Predicting clinical parameters (edema)
Won et al.	Quality assessment of meningioma radiomics studies: Bridging the gap between exploratory research and clinical applications	2021	No original article (review)
Ma et al.	Non-Invasive Radiomics Approach Predict Invasiveness of Adamantinomatous Craniopharyngioma Before Surgery	2021	Predicting clinical parameters (invasiveness)
Ugga et al.	Meningioma MRI radiomics and machine learning: systematic review, quality score assessment, and meta-analysis.	2021	No original article (review)
Shahrestani et al.	Neural network modeling for prediction of recurrence, progression, and hormonal non-remission in patients following resection of functional pituitary adenomas	2021	Predicting clinical parameters (recurrence, progression, hormonal non-remission)
Langenhuizen et al.	Radiomics-Based Prediction of Long-Term Treatment Response of Vestibular Schwannomas Following Stereotactic Radiosurgery	2021	Predicting clinical parameters (response)
Dang et al.	Investigating Predictors of Increased Length of Stay After Resection of Vestibular Schwannoma Using Machine Learning	2021	Predicting clinical parameters (length of stay after resection)
Zhang et al.	Radiomics Approach for Prediction of Recurrence in Non-Functioning Pituitary Macroadenomas	2021	Predicting clinical parameters (recurrence)s
Chen et al.	The value of conventional magnetic resonance imaging based radiomic model in predicting the texture of pituitary macroadenoma	2020	Predicting clinical parameters (consistency); also published in Chinese
Cepeda et al.	Meningioma Consistency Can Be Defined by Combining the Radiomic Features of Magnetic Resonance Imaging and Ultrasound	2020	Predicting clinical parameters (consistency)

	Elastography. A Pilot Study Using Machine Learning Classifiers		
Han et al.	Meningiomas: Preoperative predictive histopathological grading based on radiomics of MRI	2021	Predicting pathologic features (grade)
Gu et al.	The Current State of Radiomics for Meningiomas: Promises and Challenges	2020	No original article (review)
Yang et al.	Prediction of pseudoprogression and long-term outcome of vestibular schwannoma after Gamma Knife radiosurgery based on preradiosurgical MR radiomics	2020	Predicting clinical parameters (pseudoprogression, response)
Kim et al.	Thin-Slice Pituitary MRI with Deep Learning-based Reconstruction: Diagnostic Performance in a Postoperative Setting.	2020	Other (image reconstruction)
Wang et al.	Differentiation of gastric schwannomas from gastrointestinal stromal tumors by CT using machine learning	2020	Other (no CNS - gastric tumors)
Kalasauskas et al.	Identification of High-Risk Atypical Meningiomas According to Semantic and Radiomic Features	2020	Predicting pathologic features (subtype)
Prince et al.	Robust deep learning classification of adamantinomatous craniopharyngioma from limited preoperative radiographic images	2020	Predicting pathologic features (subtype)
Neromyliotis	Machine Learning in Meningioma MRI: Past to Present. A Narrative Review.	2020	No original article (review)
Zhang et al.	Radiomic features of magnetic resonance images as novel preoperative predictive factors of bone invasion in meningiomas	2020	Predicting clinical parameters (bone invasion)
Zhao et al.	The Evaluation of Radiomic Models in Distinguishing Pilocytic Astrocytoma From Cystic Oligodendroglioma With Multiparametric MRI	2020	Predicting pathologic features (subtype)
Kandemirli et al.	Presurgical detection of brain invasion status in meningiomas based on first-order histogram based texture analysis of contrast enhanced imaging	2020	Predicting clinical parameters (brain invasion)
Hu et al.	Machine learning-based radiomics analysis in predicting the meningioma grade using multiparametric MRI	2020	Predicting pathologic features (grade)
Machado et al.	MRI radiomics for the prediction of recurrence in patients with clinically non-functioning pituitary macroadenomas	2020	Predicting clinical parameters (recurrence)
Park et al.	Radiomics model predicts granulation pattern in growth hormone-secreting pituitary adenomas	2020	Predicting pathologic features (granulation pattern)
Zhang et al.	A radiomics model for preoperative prediction of brain invasion in meningioma non-invasively based on MRI: A multicentre study	2020	Predicting clinical parameters (brain invasion)

Soldozy et al.	Pituitary Tumors in the Computational Era, Exploring Novel Approaches to Diagnosis, and Outcome Prediction with Machine Learning.	2020	No original article (review)
Cuocolo et al.	Prediction of pituitary adenoma surgical consistency: radiomic data mining and machine learning on T2-weighted MRI	2020	Predicting clinical parameters (consistency)
McGrath et al.	Manual segmentation versus semi-automated segmentation for quantifying vestibular schwannoma volume on MRI	2020	Semi-automatic segmentation
Bi et al.	Differentiate cavernous hemangioma from schwannoma with artificial intelligence (AI)	2020	Predicting pathologic features (entity)
Liu et al.	Preoperative vascular heterogeneity and aggressiveness assessment of pituitary macroadenoma based on dynamic contrast-enhanced MRI texture analysis	2020	Predicting clinical parameters (vascular heterogeneity and aggressiveness)
Khayat Kashani et al.	Prediction value of preoperative findings on meningioma grading using artificial neural network	2020	Predicting pathologic features (grade)
Voglis et al.	Feasibility of machine learning based predictive modelling of postoperative hyponatremia after pituitary surgery	2020	Predicting clinical parameters (hyponatremia)
Chu et al.	A radiomics model for preoperative prediction of brain invasion in meningioma non-invasively based on MRI: A multicentre study	2020	Predicting pathologic features (grade)
Cha et al.	Machine learning approach for prediction of hearing preservation in vestibular schwannoma surgery	2020	Predicting clinical parameters (hearing preservation)
Abouzari et al.	Prediction of vestibular schwannoma recurrence using artificial neural network	2020	Predicting clinical parameters (recurrence)
Li et al.	Ependymoma and pilocytic astrocytoma: Differentiation using radiomics approach based on machine learning	2020	Predicting pathologic features (entity)
Goertz et al.	Single-Step Resection of Sphenoorbital Meningiomas and Orbital Reconstruction Using Customized CAD/CAM Implants	2020	No ML (Computer-aided design of implants)
Tian et al.	Radiomic Analysis of Craniopharyngioma and Meningioma in the Sellar/Parasellar Area with MR Images Features and Texture Features: A Feasible Study	2020	Predicting pathologic features (entity)
van Staalduinen et al.	Editorial for "Texture Analysis of High b-value Diffusion-Weighted Imaging for Evaluating Consistency of Pituitary Macroadenomas"	2020	No original article (editorial)
Peng et al.	A machine learning model to precisely immunohistochemically classify pituitary adenoma subtypes with radiomics based on preoperative magnetic resonance imaging	2020	Predicting pathologic features (subtype)
McCradden et al.	Ethical concerns around use of artificial intelligence in health care research from the	2020	No ML

	perspective of patients with meningioma, caregivers and health care providers: a qualitative study		
Saha et al.	Machine learning applications in imaging analysis for patients with pituitary tumors: a review of the current literature and future directions	2020	No original article (review)
Chen et al.	The Diagnostic Value of Radiomics-Based Machine Learning in Predicting the Grade of Meningiomas Using Conventional Magnetic Resonance Imaging: A Preliminary Study	2020	Predicting pathologic features (grade)
Maki et al.	A Deep Convolutional Neural Network With Performance Comparable to Radiologists for Differentiating Between Spinal Schwannoma and Meningioma	2020	Predicting pathologic features (entity)
Su et al.	Texture Analysis of High b-Value Diffusion-Weighted Imaging for Evaluating Consistency of Pituitary Macroadenomas	2020	Predicting clinical parameters (consistency)
Ke et al.	Differentiation Between Benign and Nonbenign Meningiomas by Using Texture Analysis From Multiparametric MRI	2020	Predicting pathologic features (grade)
Zhu et al.	Automatic Prediction of Meningioma Grade Image Based on Data Amplification and Improved Convolutional Neural Network	2019	Predicting pathologic features (grade)
Morin et al.	Integrated models incorporating radiologic and radiomic features predict meningioma grade, local failure, and overall survival	2019	Predicting pathologic features (grade)
Fan et al.	Development and validation of an MRI-based radiomic signature for the preoperative prediction of treatment response in patients with invasive functional pituitary adenoma	2019	Predicting clinical parameters (treatment response)
Speckter et al.	Texture Analysis of Standard Magnetic Resonance Images to Predict Response to Gamma Knife Radiosurgery in Vestibular Schwannomas	2019	Predicting clinical parameters (treatment response)
Laukamp et al.	Accuracy of Radiomics-Based Feature Analysis on Multiparametric Magnetic Resonance Images for Noninvasive Meningioma Grading	2019	Predicting pathologic features (grade)
Hamerla et al.	Comparison of machine learning classifiers for differentiation of grade 1 from higher gradings in meningioma: A multicenter radiomics study	2019	Predicting pathologic features (grade)
Mekki et al.	Machine learning defined diagnostic criteria for differentiating pituitary metastasis from autoimmune hypophysitis in patients undergoing immune checkpoint blockade therapy	2019	Predicting clinical parameters (tumor vs. inflammation)
Lovo et al.	Automated Stereotactic Gamma Ray Radiosurgery to the Pituitary Gland in	2019	No ML

	Terminally Ill Cancer Patients with Opioid Refractory Pain		
Ugga et al.	Prediction of high proliferative index in pituitary macroadenomas using MRI-based radiomics and machine learning	2019	Predicting pathologic features (proliferative index)
Zhang et al.	Radiomics approach for prediction of recurrence in skull base meningiomas	2019	Predicting clinical parameters (recurrence)
Staartjes et al.	Neural network-based identification of patients at high risk for intraoperative cerebrospinal fluid leaks in endoscopic pituitary surgery	2019	Predicting clinical parameters (risk of cerebrospinal fluid leak)
Li et al.	Presurgical differentiation between malignant haemangiopericytoma and angiomatous meningioma by a radiomics approach based on texture analysis	2019	Predicting pathologic features (type)
Zhu et al.	A deep learning radiomics model for preoperative grading in meningioma	2019	Predicting pathologic features (grade)
Zeynalova et al.	Preoperative evaluation of tumour consistency in pituitary macroadenomas: a machine learning-based histogram analysis on conventional T2-weighted MRI	2019	Predicting clinical parameters (consistency)
Banzato et al.	Accuracy of deep learning to differentiate the histopathological grading of meningiomas on MR images: A preliminary study	2019	Predicting pathologic features (grade)
Niu et al.	Differentiation Researches on the Meningioma Subtypes by Radiomics from Contrast-Enhanced Magnetic Resonance Imaging: A Preliminary Study	2019	Predicting pathologic features (subtype)
Chen et al.	Differentiation Researches on the Meningioma Subtypes by Radiomics from Contrast-Enhanced Magnetic Resonance Imaging: A Preliminary Study	2019	Predicting pathologic features (entity)
Rui et al.	MR textural analysis on contrast enhanced 3D-SPACE images in assessment of consistency of pituitary macroadenoma	2019	Predicting clinical parameters (consistency)
Speckter et al.	Pretreatment texture analysis of routine MR images and shape analysis of the diffusion tensor for prediction of volumetric response after radiosurgery for meningioma	2018	Predicting clinical parameters (treatment response)
Hollon et al.	A machine learning approach to predict early outcomes after pituitary adenoma surgery	2018	Predicting clinical parameters (outcome)
Hale et al.	Machine learning analyses can differentiate meningioma grade by features on magnetic resonance imaging	2018	Predicting pathologic features (grade)
Park et al.	Radiomics and machine learning may accurately predict the grade and histological subtype in meningiomas using conventional and diffusion tensor imaging	2018	Predicting pathologic features (grade)

Dong et al.	Differentiation between pilocytic astrocytoma and glioblastoma: a decision tree model using contrast-enhanced magnetic resonance imaging-derived quantitative radiomic features	2018	Predicting pathologic features (entity)
Banzato et al.	A methodological approach for deep learning to distinguish between meningiomas and gliomas on canine MR-images	2018	Animal (dog)
Niu et al.	Preoperative prediction of cavernous sinus invasion by pituitary adenomas using a radiomics method based on magnetic resonance images	2018	Predicting clinical parameters (cavernous sinus invasion)
Kanazawa et al.	Preoperative Prediction of Solitary Fibrous Tumor/Hemangiopericytoma and Angiomatous Meningioma Using Magnetic Resonance Imaging Texture Analysis	2018	Predicting pathologic features (entity)
Muhlestein et al.	Machine learning ensemble models predict total charges and drivers of cost for transsphenoidal surgery for pituitary tumor	2018	Other (predicting drivers of cost)
Banzato et al.	Development of a deep convolutional neural network to predict grading of canine meningiomas from magnetic resonance images	2018	Animal (dog)
Lu et al.	The diagnostic value of texture analysis in predicting WHO grades of meningiomas based on ADC maps: an attempt using decision tree and decision forest	2018	Predicting pathologic features (grade)
Galm et al.	MRI texture analysis as a predictor of tumor recurrence or progression in patients with clinically non-functioning pituitary adenomas	2018	Predicting clinical parameters (recurrence)
Muhlestein et al.	Using a Guided Machine Learning Ensemble Model to Predict Discharge Disposition following Meningioma Resection	2018	Predicting clinical parameters (discharge disposition)
MacKeith et al.	A Comparison of Repeatability and Usability of Semi-Automated Volume Segmentation Tools for Measurement of Vestibular Schwannomas	2018	Semi-automatic segmentation
Zhang et al.	Non-invasive radiomics approach potentially predicts non-functioning pituitary adenomas subtypes before surgery	2018	Predicting pathologic features (subtype)
Lenz et al.	Automated differentiation between meningioma and healthy brain tissue based on optical coherence tomography ex vivo images using texture features	2018	Other (no radiology data, tissue)
MacKeith et al.	A comparison of semi-automated volumetric vs linear measurement of small vestibular schwannomas	2018	Semi-automatic segmentation
Coroller et al.	Radiographic prediction of meningioma grade by semantic and radiomic features	2017	Predicting pathologic features (grade)

Chavali et al.	Meningiomas: Objective assessment of proliferative indices by immunohistochemistry and automated counting method	2017	Other (no radiology data, tissue)
Jaiswal et al.	Meningiomas: Objective assessment of proliferative indices by immunohistochemistry and automated counting method	2017	Other (no radiology data, tissue)
Banzato et al.	Texture analysis of magnetic resonance images to predict histologic grade of meningiomas in dogs	2017	Animal (dog)
Carolus et al.	One-step CAD/CAM titanium cranioplasty after drilling template-assisted resection of intraosseous skull base meningioma: technical note	2017	No ML (Computer-aided design of implants)
Qiao et al.	Comparison of multifocal visual evoked potential, static automated perimetry, and optical coherence tomography findings for assessing visual pathways in patients with pituitary adenomas	2015	No ML
Garrido et al.	Quantitative histological assessment of xenobiotic-induced liver enzyme induction and pituitary-thyroid axis stimulation in rats using whole-slide automated image analysis	2013	No ML
Chang et al.	Computer-aided volumetric analysis as a sensitive tool for the management of incidental meningiomas	2012	No ML
Brossaud et al.	Use of an automated ACTH assay for the diagnosis of pituitary and adrenal-related diseases	2011	No ML
Gorzalka et al.	Integration of endocannabinoid signaling into the neural network regulating stress-induced activation of the hypothalamic-pituitary-adrenal axis	2009	No ML
Grala et al.	New automated image analysis method for the assessment of Ki-67 labeling index in meningiomas	2009	No ML
Kim et al.	Automated nuclear segmentation in the determination of the Ki-67 labeling index in meningiomas	2006	No ML
Pillay et al.	Computer-aided/image-guided and video-endoscopic resection of pituitary tumors	2000	No ML
